# A Novel Embolization Technique to Stem Hemorrhage Complications and Cancer

**DOI:** 10.4274/balkanmedj.galenos.2020.2020.3.109

**Published:** 2020-06-01

**Authors:** Undurti N Das

**Affiliations:** 1 R&D, UND Life Sciences, WA, United States; 2 Department of Medicine, GVP Medical College and Hospital, BioScience Research Centrer, India

Transcatheter coil or medical glue embolization is one of the most effective treatment options for various bleeding complications that may occur during various transcatheter procedures, such as coronary artery catheterization and bleeding that occur during percutaneous nephrolithotomy (PCNL) treatment. Various other materials that could be used in embolization include coils, ethanol, sodium tetradecyl sulfate, cyanoacrylate, polyvinyl alcohol, microspheres, and gelatin sponge (Gelfoam), among others. Embolic agents are either temporary or permanent. Permanent agents are more common, and there are many applicable subsets, including liquid agents, particulates, coils, and detachable plugs and balloons (Figure 1).

Catheter embolization can be applied to almost any part of the body to control or prevent abnormal bleeding due to several causes (1). Some common problems that are treated using catheter embolization include:

i. Bleeding due to traumatic injury. Most abdominal and pelvic injuries that cause bleeding because of ruptured arteries can be controlled by embolization. These injuries are generally due to motor vehicle accidents.

ii. Gastrointestinal tract diseases, such as ulcers or diverticular disease that result in bleeding can be treated by embolization, and are the first line of treatment for gastrointestinal bleeding of any cause.

iii. Bleeding, as a result of vascular malformations, such as arteriovenous malformations in the lungs or brain, is treated by catheter embolization.

iv. Embolization is used to treat tumor bleeding.

• Menorrhagia, or heavy or prolonged menstrual bleeding due to uterine fibroids, can be treated by embolization as an alternative to hysterectomy.

• Tumor embolization to decrease or cut off the blood supply to tumors is another good indication for catheter embolization. This technique is used when the tumor is difficult to reach or too vascular or impossible to remove. The embolization procedure may also be used to administer chemotherapy. Tumor reduction that occurs following chemoembolization may help to shrink a tumor, thereby enabling its removal surgically.

• Elimination of an arteriovenous malformation or arteriovenous fistula or aneurysm, especially in the brain or spinal cord, is another excellent indication for embolization.

• Treatment of varicoceles in the scrotum by embolization is also not uncommon.

• Decreasing the size of congenital venous malformations to decrease pain, swelling, and clot formation can also be achieved by embolization.

## Subcutaneous fat occludes hemorrhagic blood vessels

Given the versatility of the embolization procedure, Ari et al. (2) utilized the subcutaneous fat tissue taken with the subcutaneous fascia from the femoral site to stop the bleeding complication from the PCNL. Using this subcutaneous fat tissue embolization method, the authors could successfully stop the bleeding in two patients. In addition, they demonstrated that the treatment option was safe and reliable for PCNL (2). The advantages of the technique used by the authors are that the embolization could be achieved quickly without the guidewire ever being moved, and the technique seems to be quickly employed to embolize the bleeding point rapidly. The risk of infection and an allergic reaction is likely to be low because it uses autologous subcutaneous tissue. Furthermore, this technique is inexpensive since it does not require any extra material regarding the materials needed to implement the technique.

## Vaso-occlusive action of lipids

**Figure 2:**
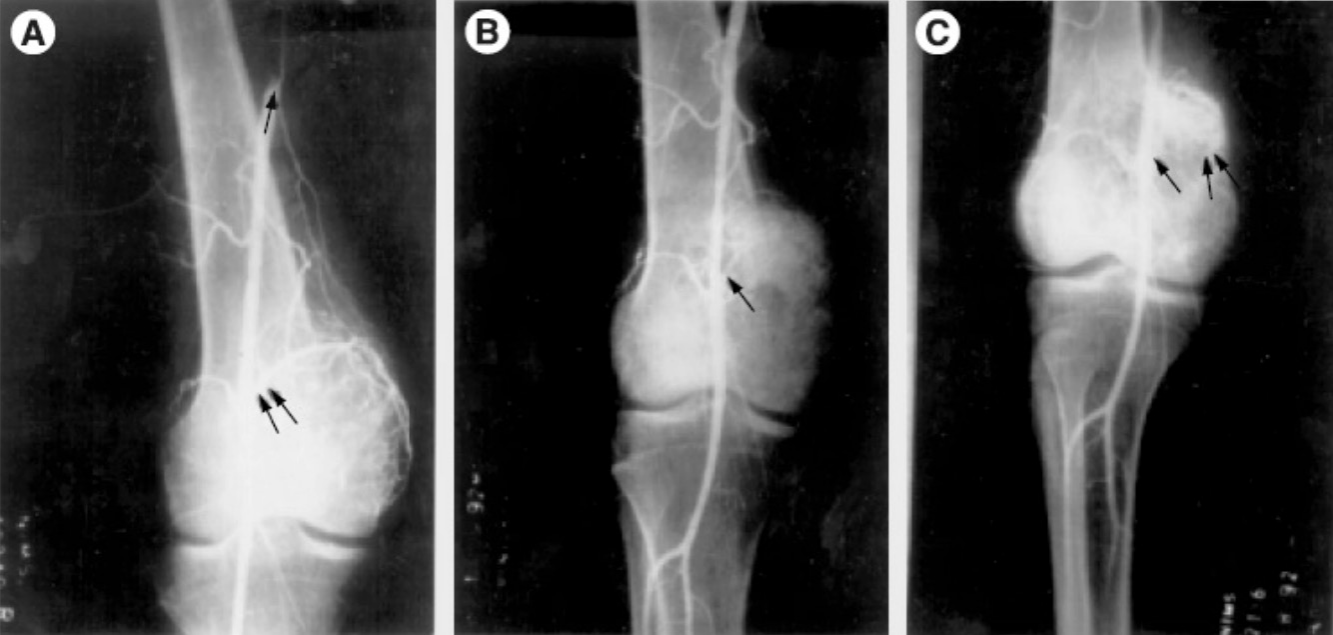
Effect of LGIOC on the tumor’s blood supply in a giant cell tumor. A. Angiogram of a patient with giant cell tumor of the right femur just before LGIOC injection. Double arrows show the origin of the tumor-feeding vessels. B. Angiogram performed immediately after the injection of LGIOC. The arrow shows the site of complete occlusion of the tumor-feeding vessel. Normal blood vessels, which were distal and in the path of the blood flow, are much smaller in diameter compared with the main tumor-feeding vessel, remained patent. C. Angiogram performed 10 days after the injection of LGIOC. The single arrow shows the site of occlusion of the main tumor-feeding vessel. Double arrows show the accumulation of LGIOC in the tumor.

**Figure 3:**
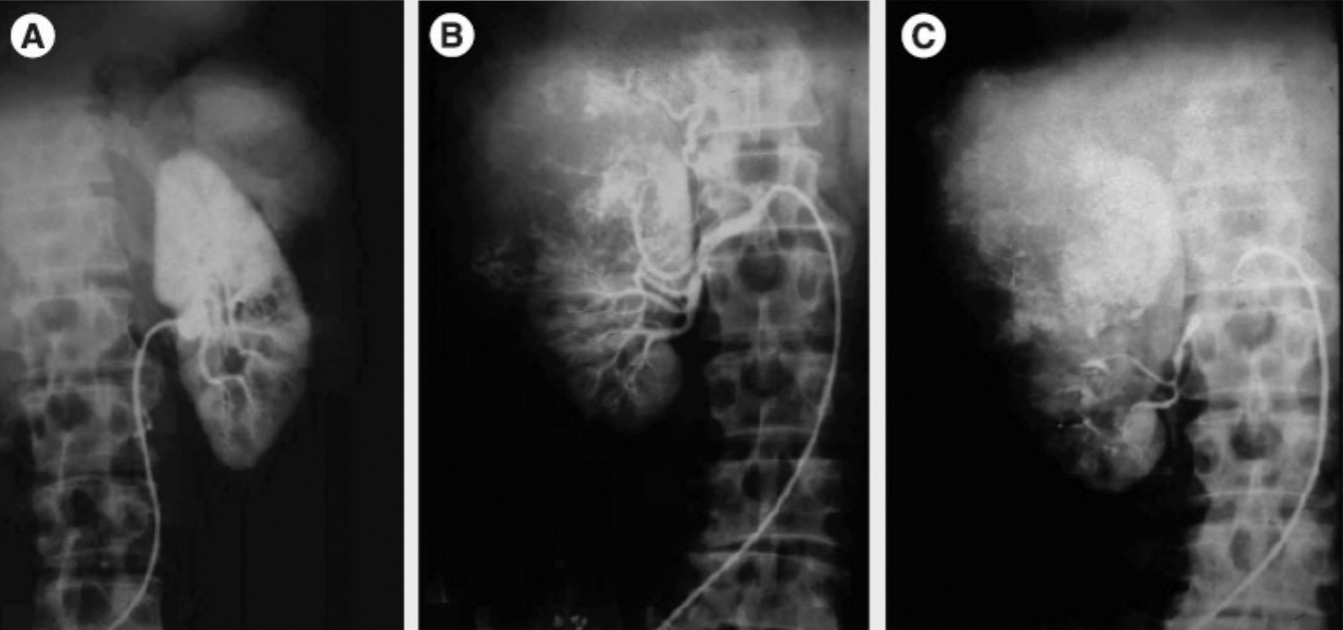
Selective occlusion of tumor-feeding vessels. A. Angiogram of the normal right kidney. B. The angiogram of the same patient shows the enlarged and distorted left kidney due to the presence of a renal tumor in the upper pole. The lateral margin of the kidney is not clear, and an increase in blood supply to the tumor can be seen. C. The angiogram of the left kidney performed immediately after the injection of LGIOC. Occlusion of the tumor-feeding vessels but not those feeding the normal lower pole of the kidney which is supplied by the two normal blood vessels, can be seen.

In this context, it is noteworthy that a similar technique was used to block only the tumor feeding vessels that resulted in the regression of the hepatoma, a giant cell tumor of the bone, and renal cell carcinoma (3,4). In this technique, we used the lithium salt of gamma-linolenic acid (GLA, 18;3 n-6), a small molecular weight lipid that has been conjugated to an iodized salt solution that is radio-opaque. This new molecule, called LGIOC (Lithium-gamma-linolenic acid conjugated to an oily lymphographic agent), when injected close to the tumor feeding vessel completely blocked all the tumor feeding vessels without any effect on normal blood vessels (Figures 2, 3). In this instance, the gamma-linolenic acid used is also a lipid (fat) but is different from the subcutaneous fat used by Ari et al. (2). It is possible that the subcutaneous fat used by Ari et al. (2) also contains some amount of gamma-linolenic acid, but this needs to be confirmed by further analysis. In general, subcutaneous fat is a mixture of several lipids, such as cholesterol, triglycerides, saturated and unsaturated fats, and other lipids. Exactly how the subcutaneous fat used by Ari et al. (2) and LGIOC employed by us can block the blood supply is not clear.

In general, when cytotoxic drugs are infused, they would cause vasospasticity leading to vasospasm that is of short duration: 24-48 hours. In our study (3,4), the occlusion of tumor-feeding vessels after the infusion of LGIOC lasted for more than 7-10 days and, in one of our patients, the occlusion of the tumor-feeding vessels lasted for more than three months, and in another, for more than 15 years suggesting that the occlusion of the vessels is almost permanent. It is possible that GLA-induced free radical generation (5,6) acts on endothelial cells of the tumor-feeding vessels and induces their occlusion and thus, produces their anti-angiogenic and anti-vascular actions. Of more than 170 transcripts expressed in the endothelium, 70 were differentially expressed, including 46 that were specifically increased in tumor-associated endothelium (7), suggesting that there are significant differences in gene expression profiles in endothelium derived from normal and tumor vessels. It is possible that LGIOC [and possibly, the subcutaneous fat tissue used by Ari et al. (2)] can stimulate some of the genes and/or receptors in the endothelium of the tumor blood vessels, which may have a role in the occlusion of the tumor vessels observed in our study.

Thus, there is seems to be a significant role for subcutaneous fat and LGIOC and similar lipids in specifically occluding abnormal vessels (tumor feeding vessels, arterio-venous malformations, or vessels that rupture and bleed during some procedures). It is possible that these abnormal vessels are weak at these bleeding spots because of abnormalities in the endothelium and vessel wall that could be exploited for therapeutic purposes.

## Figures and Tables

**Figure 1:**
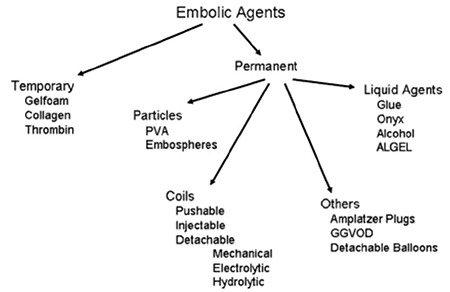
Divisions of embolic agents. GGVOD: Grifka-Gianturco vascular occlusion device, PVA: polyvinyl alcohol
